# Pseudoaneurysm of the left ventricle following apical approach TAVI

**DOI:** 10.1186/1532-429X-13-79

**Published:** 2011-12-12

**Authors:** Andrew P Vanezis, Mirza K Baig, Ian M Mitchel, Matloob Shajar, Surendra K Naik, Robert A Henderson, Thomas Mathew

**Affiliations:** 1 Department of Cardiovascular Sciences, University of Leicester, Clinical Sciences Wing, Glenfield General Hospital, Groby Road, Leicester, UK. LE3 9QP; 2 Trent Cardiac Centre, Nottingham University Hospital, Hucknall Road, Nottingham, UK. NG5 1PB

## Abstract

Symptomatic severe aortic stenosis carries a two year survival of only 50%. However many patients are unsuitable for conventional aortic valve replacement as they are considered too high risk due to significant co-morbidities. Transcatheter Aortic Valve Implantation (TAVI) offers a viable alternative for this high risk patient group, either by the femoral or apical route. This article reports a case of a pseudoaneurysm of the left ventricle following an apical approach TAVI in an elderly lady with severe aortic stenosis. To our knowledge pseduoaneuryms of the left ventricle have been reported infrequently in the literature and has yet to be established as a recognised complication of TAVI.

## Background

Symptomatic severe aortic stenosis carries a two year survival of only 50%. However many patients are unsuitable for conventional aortic valve replacement as they are considered too high risk due to significant co-morbidities. Transcatheter Aortic Valve Implantation (TAVI) offers a viable alternate for this high risk patient group, either by the femoral or apical route. The procedure was first described in 2002 by Cribier *et al *[1] and several registries have subsequently been established that indicate a procedural success rate of over 90%. This has allowed TAVI to become a viable treatment of aortic stenosis in a carefully selected group of patients where the risks of conventional surgery are thought to be too high[2-4].

This article reports a case of a pseudoaneurysm of the left ventricle (LV) following an apical approach TAVI in an elderly lady with severe aortic stenosis. To our knowledge pseduoaneuryms of the left ventricle have been reported only infrequently in the literature and it has yet to be established as a recognised complication of TAVI [5,6].

## Case Presentation

We report the case of an 86 year old British Caucasian lady with severe aortic stenosis (peak gradient 62 mmHg, mean gradient 32 mmHg, valve area 0.4 cm^2^) who described worsening dyspnoea and chest discomfort on minimal exertion. Her past medical history included coronary artery bypass grafting, hypertension and diabetes mellitus type 2.

She was deemed too high risk for conventional surgery and therefore underwent transapical approach TAVI with the implantation of a 23 mm Edwards Scientific Sapien XT valve prosthesis (a bovine tissue valve inserted on a cobalt chromium frame). Immediate transoephageal echocardiography and fluoroscopic imaging demonstrated excellent seating of the valve, subsequently confirmed angiographically.

Two days later the patient developed isolated electrocardiographic evidence of pericarditis with minimal associated chest pain. Transthoracic echocardiography demonstrated a well seated aortic prosthesis and a 0.8 cm pericardial effusion with no tamponade. The effusion was not drained and her electrocardiographic changes settled over the next few days. Three months later, transthoracic echocardiography showed an abnormal bidirectional signal at the apex (Additional File 1). Peak velocity was 139.4 cm^-1^. Cardiovascular magnetic resonance (CMR) showed a discrete pseudoaneurysm with late gadolinium myocardial enhancement (Additional Files 2 and 3). The decision was made to treat her conservatively and keep her under regular surveillance.

## Conclusions

The 30 day mortality of tranapical approach TAVI is reported by various registries at just over 10% [3,4]. Well recognised complications of TAVI via the transapical approach include bleeding, cerebral vascular events and septal haematomas. However there are few reports of pseudoaneuryms and this case helps to reinforce this outcome as a recognised complication of this procedure.

## Consent

Written informed consent was obtained from the patient for the publication of this case report and accompanying images. A copy of the written consent is available for review by the Editor-in-Chief of this journal.

## Abbreviations

CMR: Cardiac Magnetic Resonance; LV: Left Ventricle; SSPF: Steady State Free Precession imaging; TAVI: Transcatheter Aortic Valve Implantation.

## Competing interests

The authors declare that they have no competing interests.

## Authors' contributions

APV was the primary author of the text. TM conceived of the report, acted as chief editor, provided the images and was the primary physician during the patient's inpatient stay. MKB, IMM, MS, SKN and RAH were involved in the patient's care as well as editing and overseeing of the text. All authors read and approved the final manuscript.

## Supplementary Material

Additional file 1 **4 chamber view transthoracic echocardiogram of the LV showing colour flow in and out of the pseudoaneurysm**. Click here for file

Additional file 2 **CMR steady state free precession (SSFP) sequence showing pseudoaneurysm of the LV apex at the incision site**. Click here for file

Additional file 3 **First pass gadolinium scan showing flow into the pseudoaneurysm**. Click here for file
